# Out-of-Hospital Cardiac Arrest Prospective Epidemiology Monitoring during the First Five Years of EuReCa Program Implementation in Serbia

**DOI:** 10.1017/S1049023X22002424

**Published:** 2023-02

**Authors:** Suzana S. Randjelovic, Srdjan S. Nikolovski, Jelena Z. Tijanic, Ivana A. Obradovic, Zoran Z. Fiser, Aleksandra D. Lazic, Violetta I. Raffay

**Affiliations:** 1. University Clinical Center Kragujevac, Kragujevac, Serbia; 2. University of Belgrade Faculty of Medicine, Belgrade, Serbia; Serbian Resuscitation Council, Novi Sad, Serbia; 3. Municipal Institute of Emergency Medicine, Kragujevac, Serbia; 4. Department of Anesthesia, Hospital “Sveti Vracevi,” Bijeljina, Bosnia and Herzegovina; 5. Municipal Institute of Emergency Medicine, Novi Sad, Serbia; 6. Serbian Resuscitation Council, Novi Sad, Serbia; Emergency Center, Clinical Center of Vojvodina, Novi Sad, Serbia; 7. European University Cyprus, School of Medicine, Department of Medicine, Nicosia, Cyprus

**Keywords:** cardiac arrest, resuscitation, return of spontaneous circulation

## Abstract

**Introduction:**

Poor outcome is still a challenging concern in patients with out-of-hospital cardiac arrest (OHCA) world-wide and there are large differences between European countries regarding not only incidence rates, but survival rates as well. In 2014, Serbian Resuscitation Council initiated regular data collection on epidemiology of OHCA, according to the European Registry of Cardiac Arrest (EuReCa) study protocol.

**Study Objective:**

The aim of this study is to analyze the results of the first five-year period after initiation of EuReCa study protocol elements implementation in OHCA epidemiological data collection in Serbia.

**Methods:**

The observed period in this study is about the data on OHCA, collected within the observed area of 16 municipalities covering 1,604,015 citizens, during the period from October 1, 2014 – December 31, 2019. The study included data on all-cause OHCA in both adult and pediatric patients, according to the EuReCa One study protocol, of which all segments were observed.

**Results:**

Within the study period, 5,196 OHCA patients were observed with annual incidence of 83.60/100,000. Of all registered events, 43.9% were witnessed. The most common collapse location was patient’s residence (88.7%). Within the group of initiated cardiopulmonary resuscitation (CPR), cardiac etiology was observed in 80.5% of cases and shockable rhythm in 21.7%. Return of spontaneous circulation (ROSC) prior to hospital admission was significantly more frequently achieved and maintained on admission in witnessed cases, cases occurring out of patient’s residence, and in cases with shockable initial rhythm (P <.01).

**Conclusion:**

The OHCA incidence in Serbia is comparable with the incidence in the majority of European countries, and survival rates are now significantly higher in Utstein events compared to previous results from Serbia. Enrolment of witnessing bystanders in initiating CPR measures remains a concern requiring effort towards understanding of CPR initiation importance and education of general population in administering CPR measures.

## Introduction

Out-of-hospital cardiac arrest (OHCA) is a serious public health issue. There are approximately 420,000 cardiac arrests annually in the United States and approximately 275,000 in Europe.^1^ Early reaction is one of the key factors influencing the outcome in these patients.^2^ According to the latest European Resuscitation Council’s (Niel, Belgium) recommendations, chain of survival includes broader general population enrollment to early recognition of cardiac arrest, early cardiopulmonary resuscitation (CPR) initiation, and early application of direct-current shock.^3,4^ The European Registry of Cardiac Arrest (EuReCa) One was the first study which collected data into a single database, from 27 countries across Europe.^5^ As such, OHCA has been monitored in the Republic of Serbia since 2014 when the inclusion of numerous Serbian health care institutions in the EuReCa One project occurred.^6^ With the presence of the collected data, it became possible to compare the obtained results for a five-year period with other European regions.

The EuReCa registry aims to determine the incidence, care process, and outcome of patients with OHCA in a large number of European countries. The final parameters involved in this prospective analysis were return of spontaneous circulation (ROSC), hospital admission, and 30-day survival.^6^

Commonly, OHCA is a health problem associated with poor outcome. Until 2014, comprehensive epidemiological OHCA data in Serbia were not collected on a regular basis. With enrollment of Serbian Resuscitation Council (Novi Sad, Serbia) in the EuReCa One study, the collection of data according to the Utstein protocol has been initiated.^7^ Initial data evaluation showed that there is a necessity for prolonged data collection in order to improve the access and care of OHCA patients. Fortunately, the continuation of the data collection was supported by the Serbian Resuscitation Council’s projects after an authorization given by the European Resuscitation Council.

Several epidemiologic reports have been published describing epidemiological situations related to OHCA in Serbia, and this is the first encompassing a five-year period of follow-up.

Therefore, the aim of this study is to present basic epidemiological data concerning OHCA in Serbia during the five-year follow-up period and to emphasize similarities and differences related to the findings of previously published papers in Serbia and other European countries.

## Methods

Data on OHCA for this prospective observational multicentric study were collected during the period from October 1, 2014 - December 31, 2019 according to the protocol of the EuReCa One study, registered at the United States National Library of Medicine’s (Bethesda, Maryland USA) registry of clinical trials under the ID number NCT02236819. According to the EuReCa One study protocol, prior to inclusion of Serbian registry into the EuReCa One study, written approval was obtained clearly describing the permission to use and transmit defined data for research purposes on an international basis. Emergency Medical Service (EMS) centers in Serbia were enrolled on a voluntary basis, which was followed by entering the data into the unique electronic database by the main investigator of each enrolled EMS center.

The study included data on all-cause OHCA in both adult and pediatric patients where EMS intervention was present, within the geographic and administrative areas covered by the EMS centers enrolled in the study.

Data confidentiality and coding were applied according to the EuReCa One study protocol, of which all segments were observed.

Available data on patient age and gender, cause, time, and location of OHCA, witnessing and CPR measures provided by witness, as well as the data on outcome were collected for all patients and included in further analysis. Descriptive statistical models were used to evaluate the incidence of OHCA per each time period, as well as to analyze the performance of CPR measures applied by witnesses and main prehospitalization outcomes. The distribution of numerical variables data was examined by Kolmogorov-Smirnov test with Lilliefors significance correction. Mean value with standard deviation (SD) and median value with interquartile range (IQR) were utilized as a representative value for all numerical variables, based on the normality of data distribution, while frequency and percent were used to describe categorical data. Incidence was calculated using the population covered and extrapolated to incidence rates per 100,000 population per year. T-test and Mann Whitney U test were used to compare means and mean ranks of numerical variables, while Chi-square test and Fisher’s exact test were used to analyze the association of categorical variables. The analysis was performed by using Statistical Product and Service Solutions package for Windows v26.0 (IBM Corp.; Armonk, New York USA).

## Results

The population covered by the EMS centers from 16 municipalities in Serbia enrolled in the study was 1,604,015 citizens, a sample representing 22.3% of the population of Serbia (the population size was determined based on the last census performed prior to study initiation).

**Figure 1.**
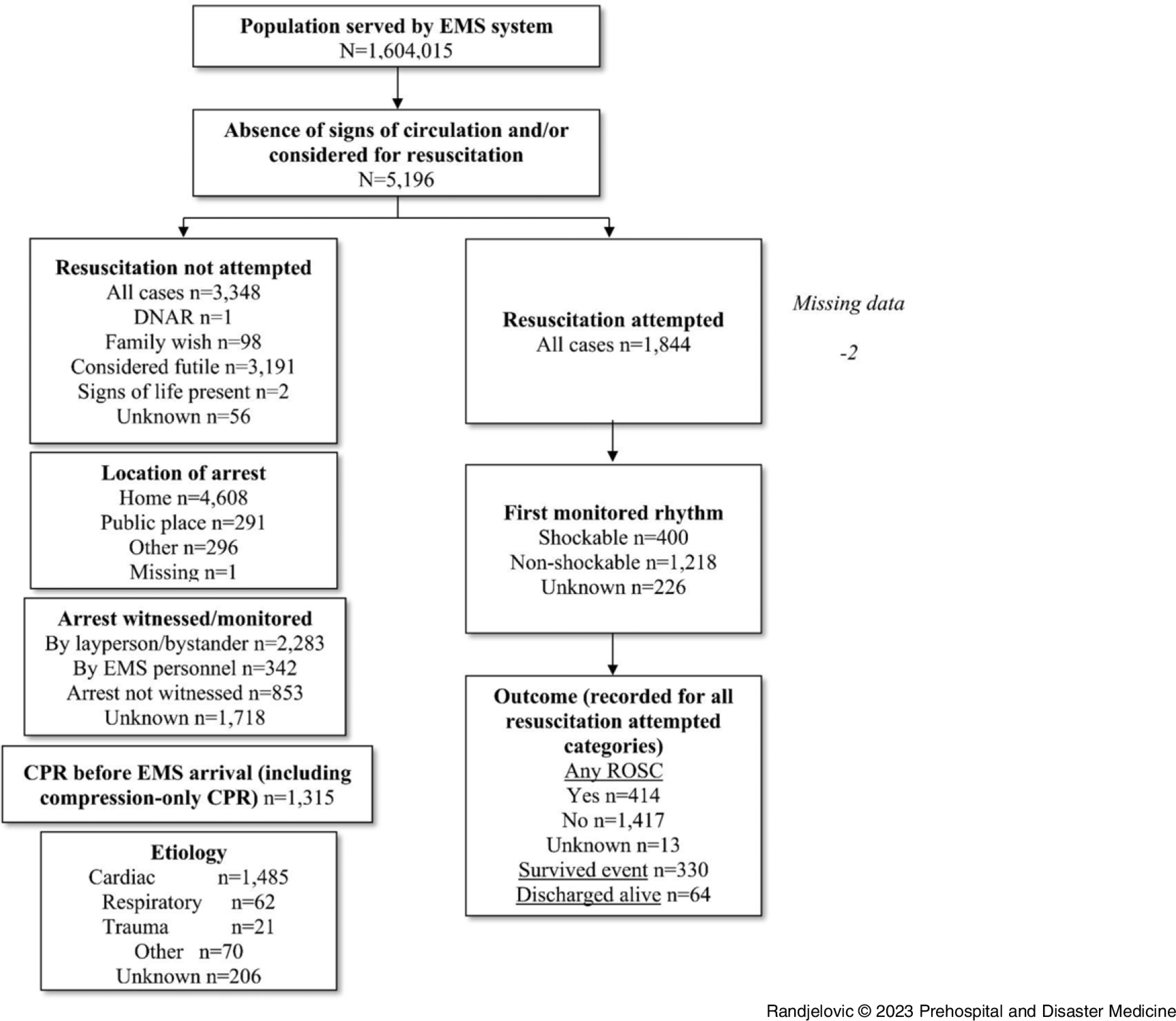
Utstein-Style Flow Diagram of Study Participants. Abbreviations: DNAR, do not attempt resuscitation; EMS, Emergency Medical Service; CPR, cardiopulmonary resuscitation; ROSC, return of spontaneous circulation.

Within the study period, 5,196 OHCA patients were registered with an annual incidence of 83.60/100,000. Of all registered OHCA patients, 2,768 were males (53.3%). Median age was 74 years (interquartile range/IQR = 63-83). Flow diagram of patients included in the study is presented in Figure 1.

**Figure 2.**
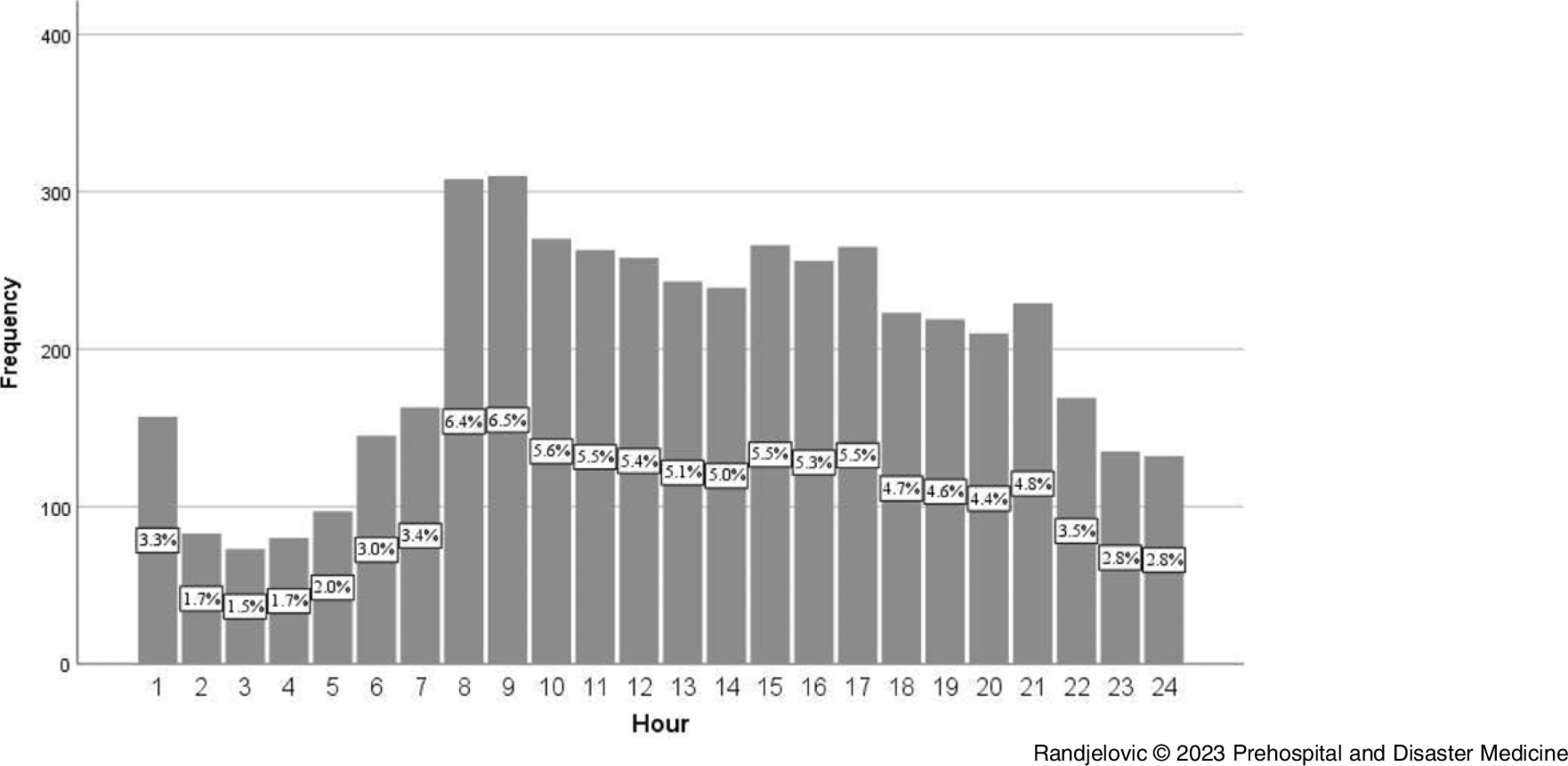
Distribution of Out-of-Hospital Cardiac Arrest Events by Each Hour per Day.

Highest frequency of OHCA (more than 6.0%) was observed between 7:00am and 9:00am, while the lowest (less than 2.0%) was between 1:00am and 5:00am (Figure 2). Less than one-half of all events (n = 2,283; 43.9%) were witnessed, and the most commonly reported location of OHCA was patient’s residence (n = 4,608; 88.7%). Within the group of initiated CPR, cardiac etiology was observed in 1,485 cases (80.5%) and shockable rhythm was achieved in 400 patients (21.7%).

Significant differences in terms of achieving ROSC at any stage (any ROSC) and admission ROSC were observed between witnessed and non-witnessed groups, residence and out-of-residence location groups, as well as shockable and non-shockable initial rhythm groups of OHCA patients, while the cardiac etiology of OHCA was not significantly associated with ROSC achievement frequency (Table 1).

**Table 1.** Between-Group Differences in Achieving Any ROSC and Admission ROSC in OHCA Patients

Variables		Any ROSC N (%)	P Value	Admission ROSC N (%)	P Value
**Witnessing OHCA**	Witnessed	388 (23.9)	<.01	314 (24.1)	<.01
	Not Witnessed	22 (12.3)		14 (10.8)	
**Etiology of OHCA**	Cardiac	264 (21.3)	.07	232 (18.7)	.03
	Non-Cardiac	33 (28.4)		31 (27.0)	
**OHCA Location**	Residence	236 (16.9)	<.01	201 (18.3)	<.01
	Out-of-Residence	178 (40.7)		129 (36.8)	
**Initial Rhythm (within CPR Attempted Group)**	Shockable	183 (45.8)	<.01	158 (43.8)	<.01
	Non-Shockable	183 (15.1)		164 (15.5)	

Abbreviations: ROSC, return of spontaneous circulation; OHCA, out-of-hospital cardiac arrest; CPR, cardiopulmonary resuscitation.

Additionally, CPR was attempted in 1,844 of OHCA patients (annual incidence: 32.49/100,000). Within that group of patients, data regarding the performer of CPR measures were present in 1,801 cases, with bystander initiating CPR in 316 cases (17.1%; 2.42/100,000 per year).

Within the bystander-CPR group, any ROSC was achieved in 266 patients (20.2%; 4.07/100,000 per year), and in the EMS-CPR group, was achieved in 122 patients (38.6%; 4.42/100,000 per year), showing highly significant difference (P <.01).

Similar finding was observed in terms of ROSC on admission, where it was achieved in the bystander-CPR group in 218 patients (16.6%; 3.34/100,000 per year) and in the EMS-CPR group in 96 patients (30.4%; 1.80/100,000 per year); P <.01.

Within the group of 2,283 bystander-witnessed events (annual incidence: 34.45/100,000) where bystander initiated CPR (n = 1,315; 71.3%; 21.24/100,000 per year), shockable initial rhythm was achieved in 283 (12.4%; 4.71/100,000 per year), any ROSC was achieved in 266 (11.7%; 4.07/100,000 per year), and ROSC on admission was achieved in 218 patients (9.5%; 3.34/100,000 per year), all cases occurring in CPR attempted group, while there were no cases with shockable rhythm, any ROSC, or admission ROSC achieved in the bystander-witnessed group where bystander did not attempt CPR.

Regarding dispatcher assistance within the group of 1,315 bystander-initiated CPR cases, data on presence/absence of EMS dispatcher assistance were present in 1,293 cases with applied CPR measures, out of which 127 (9.7%; 2.04/100,000 per year) were performed with dispatcher assistance. Significantly higher frequency of occurrence of shockable initial rhythm, any ROSC, and ROSC on admission was achieved in the dispatcher-assisted CPR group compared to the non-dispatcher-assisted CPR group (42.3% versus 22.6%, P <.01; 38.1% versus 18.5%, P <.01; and 39.4% versus 18.7%, P <.01, respectively).

Distribution of any ROSC and admission ROSC within shockable and non-shockable groups is presented in Table 2, while the annual incidence of outcome events per 100,000 citizens is presented in Table 3.

**Table 2.** ROSC and Survival on Hospital Admission of Patients with Attempted Resuscitation

	All			
	All	First Recorded Rhythm		Missing Data
		Shockable	Non-Shockable	
N	1,844	400	1,218	226
Any ROSC	414 (22.5%)	183 (45.8%)	183 (15.0%)	48
ROSC on Admission	330 (17.9%)	158 (39.5%)	164 (13.5%)	8

	**Bystander Witnessed and Cardiac Etiology**			
	All	First Recorded Rhythm		All
		Shockable	Non-Shockable	
N	920	228	682	10
Any ROSC	179 (19.5%)	98 (43.0%)	77 (11.3%)	4
ROSC on Admission	158 (17.2%)	86 (37.7%)	68 (10.0%)	4

Abbreviation: ROSC, return of spontaneous circulation.

**Table 3.** Annual Frequency and Incidence of Outcome Events

Variable	N	%	Per 100,000
OHCA	5,196	–	83.60
Residence as OHCA Location	4,608	88.7	73.17
All Witnessed OHCA	2,625	49.6	42.63
Bystander-Witnessed OHCA	2,283	43.9	34.45
EMS-Witnessed OHCA	342	6.6	2.65
CPR Attempted in Group of Witnessed All-Cause OHCA Cases	1,844	70.2	32.49
Bystander-CPR in Group of CPR Attempted All-Cause OHCA Cases	316	17.1	2.42
EMS-CPR in Group of CPR Attempted All-Cause OHCA Cases	1,315	71.3	22.24
CPR Attempted in Group of Witnessed Cardiac-Cause OHCA Cases	1,246	55.1	8.79
CPR Attempted in Group of Witnessed Non-Cardiac-Cause OHCA Cases	116	17.9	1.06
Any ROSC Achieved in Group of CPR Attempted All-Cause OHCA Cases	414	22.5	7.01
Any ROSC Achieved in Group of CPR Attempted Cardiac-Cause OHCA Cases	264	21.2	4.64
Any ROSC Achieved in Group of CPR Attempted Non-Cardiac-Cause OHCA Cases	33	28.4	2.06
Admission ROSC Achieved in Group of CPR Attempted All-Cause OHCA Cases	330	17.9	5.56

Abbreviations: OHCA, out-of-hospital cardiac arrest; EMS, Emergency Medical Service; CPR, cardiopulmonary resuscitation; ROSC, return of spontaneous circulation.

Gender was significantly associated with the occurrence of shockable initial rhythm (P <.01), where it was more commonly observed within males (7.4%) compared to the female group (3.1%). Significant differences were not observed with regard to any ROSC and admission ROSC, although slightly higher occurrence of both of those positive outcomes was observed in males (8.7% versus 5.1%, P = .423 and 5.5% versus 3.5%, P = .908, respectively).

**Figure 3.**
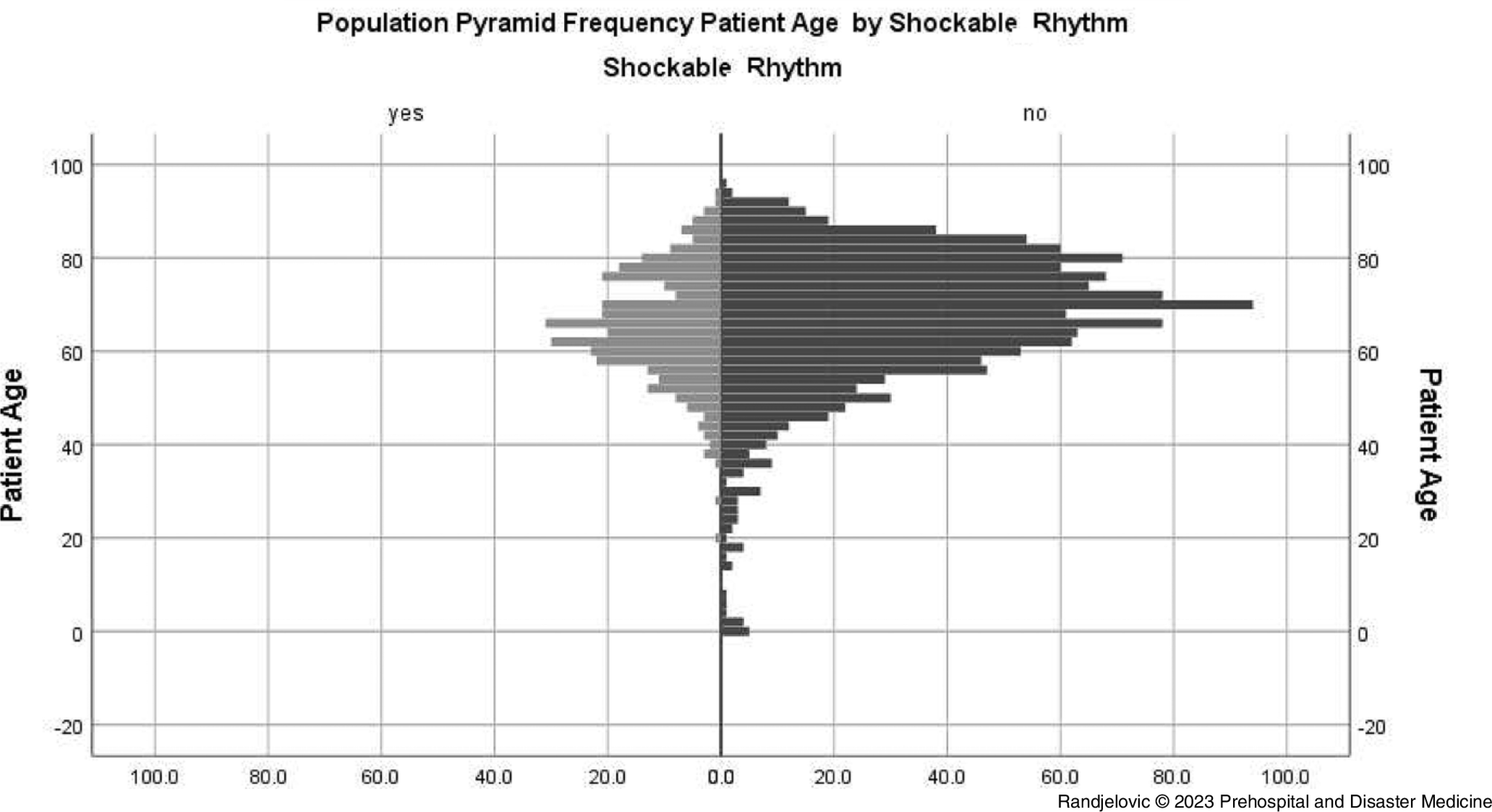
Pyramid Frequency Graph of Out-of-Hospital Cardiac Arrest Events with Attempted Cardiopulmonary Resuscitation by Patient Age in Shockable and Non-Shockable Rhythm Groups.

**Figure 4.**
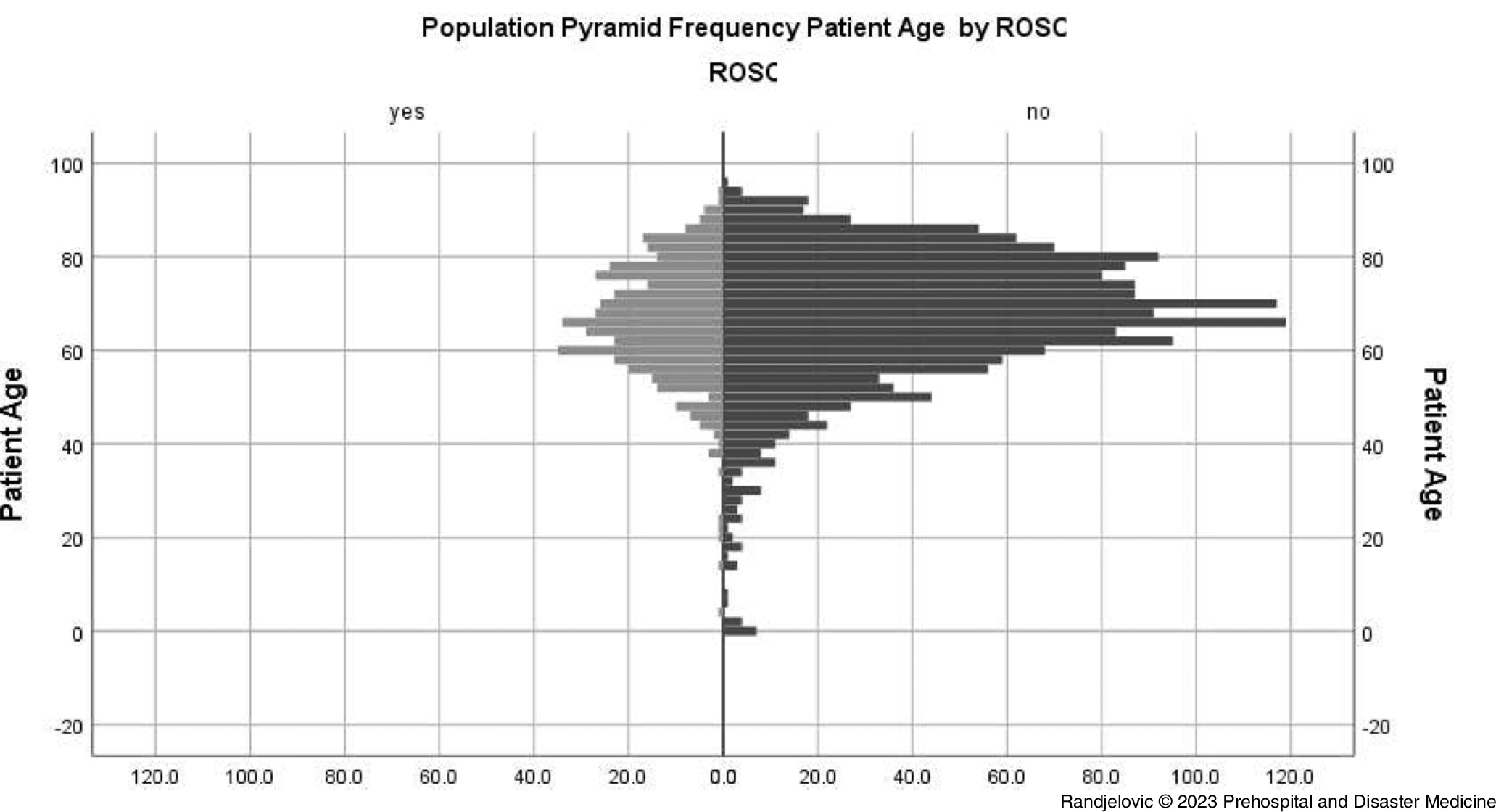
Pyramid Frequency Graph of Out-of-Hospital Cardiac Arrest Events with Attempted Cardiopulmonary Resuscitation by Patient Age in Groups With and Without Any Return of Spontaneous Circulation Achieved.

Younger age of patients was significantly associated with presence of shockable initial rhythm (median 65 years [interquartile range/IQR = 58-74] versus median 69 years [IQR = 59-77] in the non-shockable initial rhythm group; P <.01) and any ROSC (median 66 years [IQR = 59-75] versus median 68 years [IQR = 59-77] in the non-any ROSC group; P = .03); Figure 3 and Figure 4.

Median time between the EMS call and the application of direct-current shock was 10 minutes (IQR = 6-17) and did not show significant association with the occurrence of any ROSC (P = .125) and ROSC on admission (P = .162).

## Discussion

Data on OHCA incidence throughout recent studies have prominent variability. According to the EuReCa One study and the Epidemiology report of the European Resuscitation Council 2021 Guidelines, OHCA annual incidence in Europe varies between 28 and 244 and 67 and 170 per 100,000 inhabitants, respectively.^4,8^ This study reports the comparable annual incidence rate of 83.6/100,000, and previous reports of OHCA incidence in individual years imply that there is significant variability not only in time, but also in geographical distribution as well.^9–11^

The results of this study are, however, lower than the incidence shown in the 2015 and 2016 reports from Serbia (160/100,000 and 122/100,000, respectively),^9,10^ but are higher than the incidence reported from the first-half of 2017 (49.5/100,000).^11^ All these reports analyzed data on all-aged patients, just like is the case in this report.

It is important to emphasize that this study analyzed data on OHCA cases which were collected according to the Utstein protocol and EuReCa project methodology.^8,12,13^ Therefore, the results of the analysis of data collected according to these guidelines and presented in this study show a significantly higher level of homogeneity compared to many recent studies in which data were not collected according to the Utstein protocol and EuReCa methodology. However, taking an example of recent Polish reports,^14–16^ the same observations showed different values in different periods of time. The 2016 study from Poland which analyzed the data from 2013 reported the OHCA frequency of 170/100,000.^14^ A slightly lower result from the same country was observed during the period 2006-2007 (156/100,000), although that analysis was performed on OHCA patients with presumed cardiac etiology only.^15^ However, the report from the same country analyzing data during 2018 presented the incidence range of 58.9-84.5/100,000 among different provinces.^16^

Lower OHCA incidences in Europe than the incidence observed in this study were previously reported in United Kingdom (53/100,000);^17^ Spain (34/100,000);^18^ France (62/100,000);^19^ and Norway (46/100,000),^20^ while higher incidences were observed in Czech Republic (230/100,000)^21^ and Italy (123/100,000).^22^ The OHCA incidence presented in this study is, however, comparable with the results presented in the Danish 2019 Registry (93/100,000)^4^ and as an average OHCA incidence in the EuReCa One study (84/100,000), although the same study reported the incidence in Serbia of 183/100,000.^8^

Results structure in mentioned reports imply that there are differences in the organization of the prehospital emergency system and its function. In countries where death is confirmed by the EMS, there is a higher OHCA incidence rate witnessed by the EMS members compared to the countries where EMS does not have a role in death confirmation. Therefore, these differences in OHCA annual incidence rate could be explained with the organization of EMS and the difference in the methodology of data collection.

According to this study’s results, OHCA occurred in patient’s residence in 88.7% of cases (73.17/100,000), which is comparable to the findings of the EuReCa One study (69.4%).^8^ Also, this result is higher than the previous reports from Serbia (43-48/100,000 and 23.3/100,000),^10,11^ as well as the 2018 report from France (75%),^19^ while comparable at the same time with some other earlier reports from Europe.^7^

The analysis of the presence of a witness in this study showed that the incidence of witnessed OHCA is 49.6% or 42.63/100,000, which is comparable to the 2015 and 2016 Serbian reports (45.5/100,000 and 42.5/100,000),^9,10^ with significant regional variations, but also significantly higher compared to the Serbian report from the first-half of 2017.^11^ Also, this finding is slightly lower than the percentage reported in the 2016 studies from Poland and United Kingdom (60% and 53%, respectively).^14,17^

Bystander-witnessing OHCA was reported in 43.9% of all OHCA events included in this study, which is lower compared to the 13-year Swiss study published in 2016 (69.0%)^23^ and the results shown in the Irish National Registry 2019 Annual Report (50.0%).^4^

However, although there is a high percent of witnessing OHCA, a small percentage of bystanders initiate CPR measures prior to the EMS arrival on scene. In the group of 1,844 witnessed all-cause OHCA cases analyzed in this study, CPR was attempted in 17.1% of cases (2.42/100,000), which is significantly lower than the one presented in Serbian reports from 2015 and 2016,^9,10^ as well as the one from the first-half of 2017.^11^ It is also lower than the incidence in Serbia reported in the EuReCa One study (60/100,000) and previous European reports ranging from 3.26% to 6.85%.^7,8,24^

The incidence of any ROSC achieved in the group of CPR attempted OHCA cases in 2015 and 2016 Serbian reports is higher compared to the result in this study of 7.01/100,000 (10.8/100,000 and 17.6/100,000, respectively),^9,10^ but the present finding is comparable to the findings from the first-half of 2017 (7.0/100,000).^11^ Also, the percentage value of the same finding in this study (22.5%) is higher than the one from the 2014 Serbian database (16.1%), but is slightly lower to the result from the 2017 database (23.7%)^25^ and the result of the Irish National Registry 2019 Annual Report.^4^ The same result this study reports is within the range of the EuReCa One and EuReCa Two study findings, but is lower than the average values those studies showed (30.6% and 29.7%, respectively).^8,12^

In this study, CPR measures were initiated in 1,844 patients. Any ROSC was achieved in 414/1,844 patients (22.5%). In the group of initially detected shockable heart rhythm, it was achieved in 45.8%, while in non-shockable rhythm group, it was achieved in 15.0% of cases. These data are comparable with data from other countries reporting that the success rate of achieving any ROSC in the Utstein event group (the presence of a shockable rhythm with initiated CPR measures) is expected to be significantly higher than for non-Utstein events (asystole, pulseless electrical activity). The results of this study are comparable to reports from developed countries.^8^ However, there is a need to be emphasized that the data from Serbia published in the EuReCa One study indicated that there was no difference in the survival of patients with first recorded shockable rhythm compared to non-shockable rhythms.^8^ Since the Utstein comparator group results are strong indicator of rescuers’ competence, those results indicated that there was significant insufficiency present concerning the training of the staff of those EMS centers enrolled in the study for the proper recognition and treatment of patients with shockable and non-shockable rhythms. The efforts to improve the quality of those areas have resulted in a significant improvement in results, as shown in this study, making them comparable with the results of developed countries.

Of the 414 patients with any ROSC, it was maintained in 330 cases where patients were admitted to the hospital with ROSC. In other words, of 1,844 CPR attempted all-cause OHCA cases, admission ROSC was achieved in 17.9% (5.56/100,000). The percentage value is lower than the majority of recently published studies in Europe^8,12,14,17,18,20,21,24^ and comparable to some rare reports.^4^ The incidence value per 100,000 is also lower compared to previous reports, including reports from Serbia and the EuReCa One study.^8–11,20^

Admission ROSC rate in groups with shockable and non-shockable initial rhythm reported in this study (39.5% and 13.5%, respectively) is also lower compared to some recent studies.^17,24^

Dispatcher assistance also led to significantly higher occurrence of shockable initial heart rhythm, any ROSC, and admission ROSC.

Male gender has been shown as an important factor with an influence on the frequency of shockable initial rhythm, where it was observed in significantly higher rate in males than in females. Also, younger age in this study was associated with significantly higher rate of both shockable initial rhythm and any ROSC.

## Limitations

Main shortcomings of this study include its observational design and, as a consequence, limited ability to report specific causalities. Also, there is a scarcity in certain areas of data collection. Besides, the voluntary nature of EMS center inclusion caries a risk of the lack of homogeneity of data, since EMS centers who entered this study were most probably the ones being ready to be enrolled and to represent their data. Therefore, there is a great probability that this study represents mainly those areas where improvements are being made on a daily basis, and where personnel potential and quality is at high level.

## Conclusion

According to the available data, one of the main conclusions of this study is that the incidence of OHCA in Serbia is comparable with the most recent findings from the majority of European countries, and that improvement has been noted in survival rate in patients with initially detected shockable heart rhythm. However, enrollment of bystanders witnessing OHCA in initiating application of CPR measures remains a concern, and it requires additional effort towards better understanding of importance of CPR and better implementation of steps in education of general population in initiating CPR on scene.
